# Inverse renormalization group based on image super-resolution using deep convolutional networks

**DOI:** 10.1038/s41598-021-88605-w

**Published:** 2021-05-05

**Authors:** Kenta Shiina, Hiroyuki Mori, Yusuke Tomita, Hwee Kuan Lee, Yutaka Okabe

**Affiliations:** 1 Department of Physics, Tokyo Metropolitan University, Hachioji, Tokyo 192-0397 Japan; 2 Bioinformatics Institute, Agency for Science, Technology and Research (A*STAR), 30 Biopolis Street, #07-01 Matrix, Singapore, 138671 Singapore; 3 College of Engineering, Shibaura Institute of Technology, Saitama, 330-8570 Japan; 4 School of Computing, National University of Singapore, 13 Computing Drive, Singapore, 117417 Singapore; 5 Singapore Eye Research Institute (SERI), 11 Third Hospital Ave, Singapore, 168751 Singapore; 6 Image and Pervasive Access Laboratory (IPAL), 1 Fusionopolis Way, #21-01 Connexis (South Tower), Singapore, 138632 Singapore

**Keywords:** Mathematics and computing, Physics

## Abstract

The inverse renormalization group is studied based on the image super-resolution using the deep convolutional neural networks. We consider the improved correlation configuration instead of spin configuration for the spin models, such as the two-dimensional Ising and three-state Potts models. We propose a block-cluster transformation as an alternative to the block-spin transformation in dealing with the improved estimators. In the framework of the dual Monte Carlo algorithm, the block-cluster transformation is regarded as a transformation in the graph degrees of freedom, whereas the block-spin transformation is that in the spin degrees of freedom. We demonstrate that the renormalized improved correlation configuration successfully reproduces the original configuration at all the temperatures by the super-resolution scheme. Using the rule of enlargement, we repeatedly make inverse renormalization procedure to generate larger correlation configurations. To connect thermodynamics, an approximate temperature rescaling is discussed. The enlarged systems generated using the super-resolution satisfy the finite-size scaling.

## Introduction

Wilson revealed that the renormalization group (RG) is a key concept in understanding critical phenomena of phase transitions^1,2^. Kadanoff’s block-spin transformation is an idea to realize renormalization in real space^3^. The combination of the RG with Monte Carlo simulation has been successfully used as the Monte Carlo RG^4–7^. The inverse operation to generate a large system, which is called an inverse RG, was proposed by Ron, Swendsen, and Brandt^8^. This approach is free of critical slowing down for large systems.

Recent developments of machine-learning-based techniques have been applied to fundamental research, such as statistical physics^9^. A technique of supervised learning for image classification was used by Carrasquilla and Melko^10^ to propose a paradigm that is complementary to the conventional approach of studying interacting spin systems. By using large datasets of spin configurations, they classified and identified a high-temperature paramagnetic phase and a low-temperature ferromagnetic phase of the two-dimensional (2D) Ising model. Shiina et al.^11^ extended and generalized this idea so as to treat various spin models including the multi-component systems and the systems with a vector order parameter. The configuration of a long-range spatial correlation was considered instead of the spin configuration itself. Not only the second-order and the first-order transitions but also the Berezinskii-Kosterlitz-Thouless (BKT) transition^12–15^ was studied.

Tomita et al.^16^ have made further progress in this approach. In the machine-learning study of the phase classification of spin models, the Fortuin–Kasteleyn (FK)^17,18^ representation-based improved estimators^19,20^ of the correlation configuration were employed as an alternative to the ordinary correlation configuration. This method of improved estimators was applied not only to the classical spin models but also to the quantum Monte Carlo simulation using the loop algorithm. They analyzed the BKT transition of the spin-1/2 quantum XY model on the square lattice.

Another application of machine-learning study for image processing is super-resolution (SR), which is a class of techniques that enhance the resolution of an imaging system. Efthymiou et al.^21^ proposed a method to increase the size of lattice spin configuration using SR, deep convolutional neural networks^22^. This study is related to the inverse RG approach^8^. At high temperatures, however, there is a problem that the noise is largely random and difficult to learn. Because a significant reduction of variance is obtained for improved estimators at high temperatures of a disordered phase^23^, the improved correlation configuration could reduce the difficulty of SR.

There have been some other proposals for the combination of neural network and RG^24–28^. The work by Efthymiou et al.^21^ is an exceptional one to investigate an inverse RG.

In this paper, we study the inverse RG of spin models based on the SR. We consider the improved estimator of the correlation configuration instead of the spin configuration. As for the renormalization process, we propose a block-cluster transformation as an alternative to a block-spin transformation. Then, we can set up an inverse RG procedure using the SR technique. The resolution of the enhanced configuration at high temperatures is much improved compared to the SR using the spin configuration. We make inverse renormalization procedure repeatedly to generate larger correlation configurations. Introducing an approximate temperature rescaling, we show the finite-size scaling (FSS)^29–31^ for the enlarged systems. For the spin models, we treat the 2D Ising model and the 2D three-state Potts model.

## Results

### Monte Carlo renormalization group

#### Block-spin transformation

We start with the RG process. To realize the RG in real space Monte Carlo simulation, Kadanoff’s block-spin transformation^3^ is conventionally used. A majority rule is employed to determine a block spin from $2 \times 2$ spins in a block, for the square-lattice Ising model, for example.

We do not consider the transformation of the Hamiltonian. Instead, we study the transformation property of the correlation. We calculate the correlation with a distance of *L*/2 as in the machine-learning study of the phase classification of spin models^11,16^, where *L* is a linear system size. This type of correlation function was used along with the generalized scheme for the probability-changing cluster algorithm^32^. For actual calculation, we treat the average value of the *x*-direction and the *y*-direction for the site-dependent correlation, that is,

1 $$\begin{aligned} g_i(L/2) = (g[s_{x_i,y_i},s_{x_i+L/2,y_i}]+g[s_{x_i,y_i},s_{x_i,y_i+L/2}])/2, \end{aligned}$$

where $g[s, s']$ denotes a spin–spin correlation between a spin pair *s* and $s'$.

**Figure 1:**
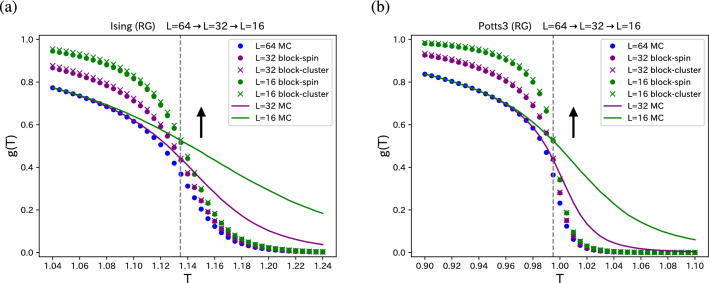
Renormalization-group procedures for (**a**) 2D Ising model and (**b**) 2D three-state Potts model. The temperature dependence of the correlation *g*(*T*) for the $64 \times 64$ systems are plotted. The results of block-spin transformation (circles) and those of block-cluster transformation (crosses) are compared. The directions of renormalization are shown by arrows for convenience. We also plot the results of $32 \times 32$ and $16 \times 16$ systems by solid curves. The exact $T_c$’s are shown by dashed line.

We performed the Monte Carlo simulation of the Ising model (2-state Potts model) on the $64 \times 64$ square lattice using the Swendsen-Wang cluster update^33^, and made block-spin transformations repeatedly. When a block-spin transformation is made one time, the linear system size becomes a half of the original size. We measured the space-averaged value of $g_i(L/2)$ for the original Ising spins and also for the block spins. We plot the temperature (*T*) dependence of the total correlation,

2 $$\begin{aligned} g(T) = \langle \frac{1}{N} \sum _{i=1}^N g_i(L/2) \rangle , \end{aligned}$$

by circles in Fig. 1a, where $N (=L \times L)$ is the system size, and the angular brackets denote the Monte Carlo average. The temperature is measured in units of the interaction *J* (in terms of the Potts model). We also plot the temperature dependence *g*(*T*) of the Ising model with $L=32$ and $L=16$ by solid curves. We observe that the block-spin correlation *g*(*T*) of $L=32$ produced from $L=64$ system and the correlation *g*(*T*) of the true $L=32$ system cross at the exact $T_c = 1/\ln (1+\sqrt{2}) = 1.1346 \cdots$, which is shown by vertical dashed line. The two-time block-spin correlation *g*(*T*) of $L=16$ and the correlation of the true $L=16$ system cross at the exact $T_c$. It is noteworthy that there are some corrections to scaling.

As another example, we treated the three-state Potts model on the square lattice. For the block-spin transformation, we use a majority rule. We plot the temperature dependence *g*(*T*) of the three-state Potts model on the square lattice with $L=64$ in Fig. 1b. The block-spin correlations are compared with the correlations of the true $L=32$ and $L=16$ systems as in the case of the Ising model, Fig. 1a. We again observe the crossing at the exact $T_c$, which is $T_c = 1/\ln (1+\sqrt{3}) = 0.9950 \cdots$ for the three-state Potts model.

#### Block-cluster transformation

In the development of the cluster update of the Monte Carlo simulation, the so-called improved estimators^19,20^ were proposed for the measurement of the correlation. In calculating spin correlations, only the spin pair belonging to the same FK cluster should be considered. In the improved estimator for the cluster representation of the *q*-state Potts model (including the Ising model), the correlation becomes 1 for the spin pair belonging to the same FK cluster, whereas it becomes 0 for the spins of different clusters. In the framework of the dual Monte Carlo algorithm^34–36^, the Markov process in the cluster update alternates between the original spin configurations (spin) and the space of the configurations of auxiliary variables (graph). Then, an improved estimator is considered to be an estimator defined in terms of the graph degrees of freedom rather than the original spin degrees of freedom.

In the proposal of using SR by Efthymiou et al.^21^, there is a problem that the noise is largely random and difficult to learn at high temperatures. We will discuss in the present paper that the improved correlation configuration solves this difficulty. However, we cannot directly apply the improved estimator for the block-spin transformation; we cannot specify the FK cluster to which a selected block spin belongs. Thus, we here propose another renormalization procedure, a block-cluster transformation. The detailed procedure of a block-cluster transformation is described in the section of Methods.

We performed the block-cluster transformation for the 2D Ising model and the three-state Potts model. The plots of the temperature dependence of the correlation *g*(*T*) are given by crosses in Fig. 1a,b. We may compare the results of the block-spin transformation (circles) and those of the block-cluster transformation (crosses). We observe that the renormalized values of *g*(*T*) are almost the same for both the Ising model and the three-state Potts model. At high temperatures fluctuations of the block-cluster transformation are smaller than those of the block-spin transformation because the improved estimators are used in the block-cluster transformation. There are very small deviations at low temperatures, which depend on the renormalization scheme. At $T_c$ of the Ising model, the block-cluster value of $g(T_c)$ for $L=32$ (purple cross) is 1.6% larger than the block-spin value (purple circle), whereas the true value of $L=32$ (purple curve) is between, and close to the value of the block-cluster transformation. From the viewpoint of transformation property of renormalization, the block-cluster transformation could be better.

### Inverse renormalization group based on super-resolution

**Figure 2:**
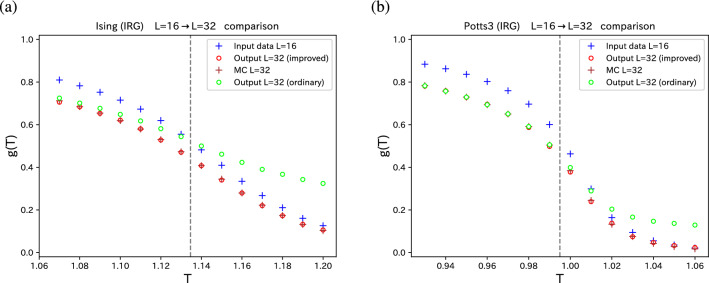
Image super-resolution for (**a**) 2D Ising model and (**b**) 2D three-state Potts model. Starting with the truncated correlation configuration ($L=16$) (blue pluses), we compare *g*(*T*) of the original configuration of test data ($L=32$) (brown pluses) with the reproduced *g*(*T*) (red circles). For comparison, we also plot the results of SR for the ordinary correlation configuration produced from the spin configuration (lime circles). The exact $T_c$’s are shown by dashed line.

#### Extension of the method of Efthymiou et al

**Figure 3:**
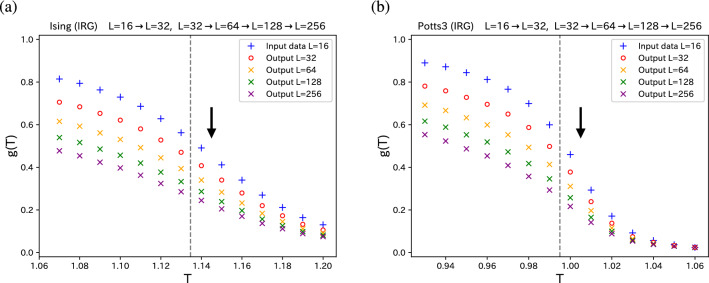
Iterative inverse renormalization-group procedures for (**a**) 2D Ising model and (**b**) 2D three-state Potts model. Starting with the truncated correlation configuration ($L=16$) (blue pluses), we obtain the reproduced *g*(*T*) of $L=32$ (red circles). The enlarged *g*(*T*)’s are plotted by orange crosses ($L=64$), green crosses ($L=128$), and purple crosses ($L=256$). The directions of inverse renormalization are shown by arrows for convenience, which are opposite to the renormalization shown in Fig. 1. The exact $T_c$’s are shown by dashed line.

We perform the inverse operation of RG, extending the method of SR with a deep convolutional neural network (CNN)^22^ due to Efthymiou et al.^21^. We use the improved estimator of the correlation configuration instead of the spin configuration. We give the detailed description of SR in the section of “Methods”. We emphasize that the present method can be applied to any *q*-state Potts model because we deal with the correlation configuration. In the case of the spin configuration, only the Ising model can be treated.

We performed the procedure of SR for the 2D Ising model and three-state Potts model. Simulating $32 \times 32$ systems, we obtain sets of original improved correlation configuration ($32 \times 32$) and the truncated improved correlation configuration ($16 \times 16$) using block-cluster transformation. For training data we use 8000 sets of configurations for each temperature. In Fig. 2, we plot the sample average of $\sum _i \xi _i$ and $\sum _i g_i$; that is, *g*(*T*) for $L=32$ (brown pluses) and $L=16$ (blue pluses). Here, $\xi _i$ and $g_i$ are original and truncated improved correlation configurations, respectively. Using the SR technique, the parameters ($\theta = (W,b)$) are tuned for each temperature. For test data we use other independent 6000 sets of original and truncated configurations. The improved correlation configuration of $L=32$ is reproduced from the $L=16$ truncated configuration using the optimized parameters $\theta$. We compare the original $\langle \sum _i \xi _i \rangle$ (brown pluses) and the reproduced $\langle \sum _i \xi _i' \rangle$ (red circles) in Fig. 2. We do not observe appreciable differences; it means that the reproduction is almost perfect for all the temperatures. In Fig. 2, for comparison, we also plot the results of SR for the ordinary correlation configuration produced from the spin configuration (lime circles). At high temperatures, there is a deviation from the true values of $L=32$ (brown pluses). To avoid this difficulty, an additional term was added in the regularization term in the loss function in Ref. ^21^. We do not need to add such an additional term for the improved estimator. The relative deviation of reproduction $\langle \sum _i \xi _i \rangle$ $\rightarrow$ $\langle \sum _i \xi _i' \rangle$ is smaller than 0.4% at all the temperatures.

Next consider the further increase of the system size. From the improved correlation configuration of $32 \times 32$ with tuned parameters $\theta$, we generate the SR configuration of $64 \times 64$. The $2L \times 2L$ output of the first SR is regarded as the input of a new network $2L \times 2L \rightarrow 4L \times 4L$. Following Efthymiou et al.^21^, we assume that the parameters (the weight matrix *W* and the bias vector *b*) are independent of the system size. In the same way, we generate $128 \times 128$ enlarged image, $256 \times 256$ enlarged image, repeatedly. In Fig. 3, we plot the iterative SR for both 2D Ising model and 2D three-state Potts model. It is noteworthy that we make Monte Carlo simulations only for the system size of $32 \times 32$. This SR procedure is a geometric procedure, and temperature has its own meaning only for $32 \times 32$ system.

**Figure 4:**
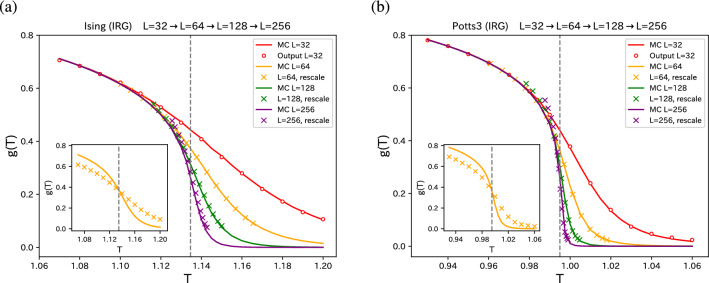
Approximate temperature rescaling for (**a**) 2D Ising model and (**b**) 2D three-state Potts model. The transformation rule ${\tilde{T}} \rightarrow T$ is established by requiring the correlation *g*(*T*) for enlarged system of $L=64$ (orange crosses) and corresponding *g*(*T*) curves of true $L=64$ system (orange curve) to collapse, which is shown in the inset of the plots in the non-rescaled *T*. In the main figure, the *g*(*T*)’s of $L=64$ (orange crosses) are plotted as a function of the rescaled *T*. By using this rule, the *g*(*T*)’s of enlarged systems of $L=128$ and $L=256$ are rescaled (crosses), and compared with the true correlations (solid curves). The exact $T_c$’s are shown by dashed line.

#### Temperature rescaling

For the one-dimensional Ising model, self-similar transformation of the Hamiltonian using the decimation scheme is possible. However, the nearest-neighbor 2D Ising model will be mapped to the model with complex interactions, such as the next-nearest-neighbor interaction, four-spin interaction, etc. Thus, for the 2D models, the transformation of Hamiltonian is not self-similar after a block-spin RG transformation, and therefore temperature alone is not sufficient to describe the coupling space of the RG configuration. We follow Efthymiou et al.^21^ to describe a method to approximate the rescaling of temperature numerically. We transform temperature such as ${\tilde{T}} = F(T)$ with the RG transformation. To avoid confusion with the activation function *f*, we here use *F* instead of *f*. In opposite direction of inverse RG transformation, transformation such as $T = F^{-1}({\tilde{T}})$ is expected. To find the rescaling, we compare the correlation *g*(*T*) calculated for enlarged system of $64 \times 64$ with the true *g*(*T*) of $64 \times 64$ system. We find the transformation ${\tilde{T}} \rightarrow T$ by requiring the corresponding *g*(*T*) curves to collapse.

Once the transformation $T = F^{-1}({\tilde{T}})$ from $32 \times 32$ to $64 \times 64$ systems is established, we use the same transformation to the inverse RG procedure of $64 \rightarrow 128$ and $128 \rightarrow 256$. In Fig. 4, we show the temperature rescaling results. The temperatures of the output of SR for $64 \times 64$ (orange crosses) are rescaled such that they collapse with the true *g*(*T*) obtained by the Monte Carlo simulation of $64 \times 64$ (orange curve), which is shown in the inset. The outputs of $128 \times 128$ and $256 \times 256$ are rescaled using the transformation $F^{-1}$ obtained by $32 \rightarrow 64$ transformation. We observe that the range of the rescaled temperature shrinks. Monte Carlo results of $128 \times 128$ and $256 \times 256$ are also given in Fig. 4 for the sake of comparison. For high temperature sides, temperature rescaling works quite well. However, for low temperature side, deviation becomes appreciable when the temperature becomes away from $T_c$. It comes from the saturation effects of *g*(*T*), that is, it approaches 1 as $T \rightarrow 0$. This phenomena often appear in FSS analysis of magnetization, for example.

For geometric inverse RG, only the Monte Carlo simulation of $32 \times 32$ system is enough. For temperature rescaling, Monte Carlo simulation of $64 \times 64$ should be added. With these small sizes of simulation, we can obtain the information on larger system sizes.

**Figure 5:**
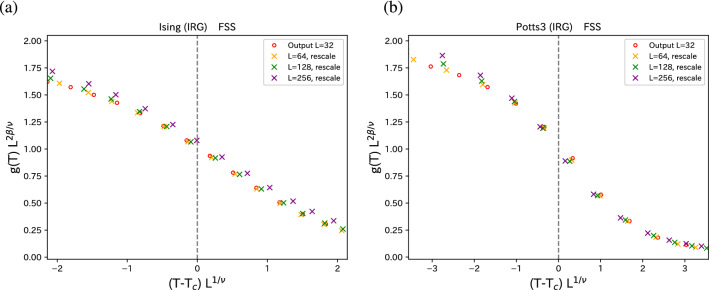
FSS for (**a**) 2D Ising model and (**b**) 2D three-state Potts model. The temperature-rescaled data shown in Fig. 4 are plotted. As for $T_c$, we used the exact values because our estimated fixed points, $T^* = F^{-1}(T^*)$, are very close to the exact values, as shown in the inset of Fig. 4. The critical exponents $2\beta /\nu$ and $1/\nu$ are chosen, such that data up to $L=128$ are collapsed. They are 0.240 (1/4) and 1.010 (1) for the Ising model, and 0.255 (4/15) and 1.215 (6/5) for the three-state Potts model. In the parentheses, the exact exponents are given.

#### Finite-size scaling

Now that we have obtained the temperature dependence *g*(*T*) for various sizes from small sizes, we try a FSS analysis^29–31^ to examine critical phenomena. The FSS function for the equation of state can be written as

3 $$\begin{aligned} g(T) L^{2\beta /\nu } = {\tilde{g}}(t L^{1/\nu }), \end{aligned}$$

where $t=T-T_c$ with the critical temperature $T_c$; $\nu$ and $\beta$ are the correlation-length and magnetization critical exponents, respectively. In Fig. 5, we show the FSS plots of the Ising model and the three-state Potts model. That is, $g(T) L^{2\beta /\nu }$ is plotted as a function of $t L^{1/\nu }$. As for the critical temperature $T_c$, we use the exact values because in the temperature rescaling transformation shown in the inset of Fig. 4, the fixed points, $T^* = F^{-1}(T^*)$, are very close to the exact values. For the critical exponents, $2\beta /\nu (= \eta )$ and $1/\nu$, best-fitted values for data collapsing up to $L=128$ were used. The chosen $2\beta /\nu$ and $1/\nu$ are 0.240 (1/4) and 1.010 (1) for the Ising model, and 0.255 (4/15) and 1.215 (6/5) for the three-state Potts model. In the parentheses, the exact exponents are given. Estimated critical exponents are $1 \sim 4 \%$ accuracy. We obtained good FSS, although the original system of small size of $L=32$ has larger corrections to FSS.

## Discussion

We have successfully realized the inverse RG based on SR approach. We have proposed the block-cluster transformation as an alternative to the block-spin transformation to use the improved estimators. We have made inverse renormalization procedure repeatedly to generate larger correlation configurations. We here make remarks on the advantage of the present method and the future directions of the research.

The advantage of using cluster representation of spin models is remarkable, and the improved estimator is quite useful. In doing so, we have introduced a block-cluster transformation, and we investigate the improved correlation configuration. The statistical advantage of improved estimator is well known^23^; at high temperatures above $T_c$, the errors for the spatial average of correlation is drastically reduced. In addition, this study elucidated the advantage in improved correlation configuration itself.

We make comments on the image as an object of image processing. Images usually have some smooth parts together with some edges. Abstract painting is not an object of image processing. A similar example is found in the case of text compression; we discuss the compression of text in natural language, while the compression of random sequences is impossible. The spin configurations have particular characteristics. There is a long-range correlation up to the correlation length. Moreover, there are some symmetries. The ordered-state spin configurations at low temperatures have some smooth parts. However, high-temperature spin configurations are random. On the other hand, improved correlation configurations have cluster structures even at high temperatures. The choice of proper “image” is important in applying the technique of image processing to the spin configuration problems.

The RG transformation is a coarse-grained procedure; thus, the truncated systems (block-spin or block-cluster transformation) have less information. Practically, it is difficult to find appropriate procedure of the inverse RG. The advantage of using the machine-learning is to find a rule to connect the renormalized configuration and the original configuration by searching for large amounts of datasets. This is the idea of the machine-learning. A single realization of $32 \times 32$ system in the present study does not have the information of larger systems. With the help of SR technique of machine-learning, we can obtain the information of larger systems which include the scaling properties.

As a preliminary study, we are also considering the block-cluster transformation for the three-dimensional (3D) systems. In the case of the simple cubic lattice, we determine a label of the block cluster from the labels of $2 \times 2 \times 2$ sites using a majority rule for the labels. The block-cluster transformation works very well for the 3D Ising model. For the 3D Ising model, a trial to improve convergence using the modified block-spin transformation^38^ in the Monte Carlo RG calculation was reported recently^39^. In the Monte Carlo RG studies^6,7,39^, the correlations between different blocking levels (*m*), (*n*), $\langle s_{\gamma }^{(n)} s_{\beta }^{(m)} \rangle - \langle s_{\gamma }^{(n)} \rangle \langle s_{\beta }^{(m)}\rangle$, are calculated. In the present formalism of block-cluster transformation, such correlations can be calculated. It will be interesting to apply the block-cluster transformation, where first-order moments $\langle s_{\beta } \rangle$ are automatically zero because of the improved estimator.

It is well-known that the wavelet transformation is a highly efficient representation of images by decomposing the image signal into high-frequency and low-frequency sub-bands. Guo et al.^40^ proposed a deep wavelet SR method to recover missing details of low-resolution images. Tomita^41^ performed the wavelet analysis of a configuration of FK clusters. The SR study on spin models using wavelet transformation will be informative.

For future studies, it will be interesting to treat continuous spin systems, such as the XY (clock) model, along the present scheme. The application of RG and inverse RG analyses to quantum systems^42^ is also challenging.

## Methods

### Detailed procedure of Block-cluster transformation

**Figure 6:**
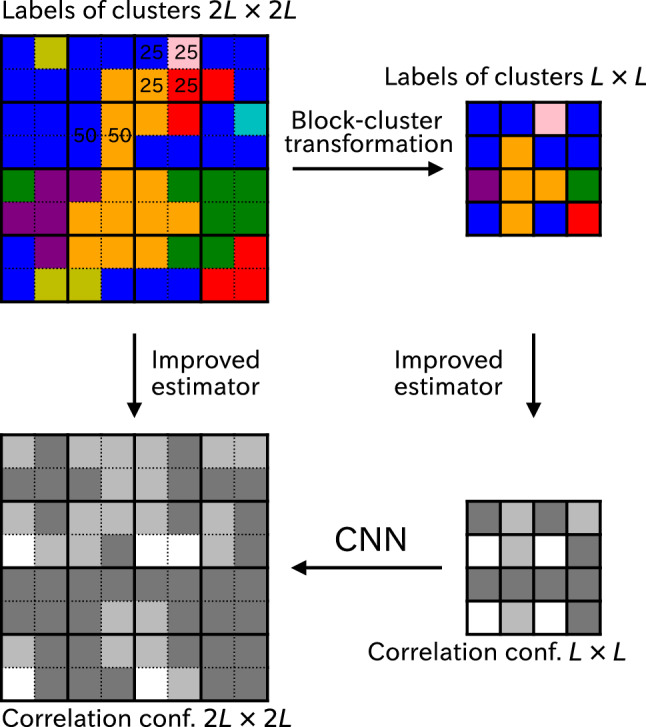
Schematic illustration of block-cluster transformation. We employ a majority rule to determine a label of the block cluster from the labels of $2 \times 2$ sites. The labels of clusters are represented by different colors. We also show the procedure of calculating improved correlation of the original configuration and the renormalized configuration together with the super-resolution process of CNN. The improved correlation configurations take a value of 1 (white), 1/2 (light gray), or 0 (dark gray).

In the cluster update of Monte Carlo simulation, the spins are classified by the FK clusters^17,18^, and we assign a label to each FK cluster. We note that the connection between the magnetization of the *q*-state Potts model and the percolation probability of the cluster model was discussed by Hu^43,44^. We employ a majority rule to determine a label of the block cluster from the labels of $2 \times 2$ sites. The procedure of the block-cluster transformation is schematically illustrated in Fig. 6, where different colors are used to assign the labels of clusters, not the spins. Even if adjacent sites have the same spins, these sites may have different colors because of the FK cluster. If the labels of two sites coincide, we take this label as the label of the block. The choice of two sites is sixfold. If there is no pair to coincide, we choose one label from four sites with 25% probability. When two sites have one label and the other two sites have another label, one may pick up one with 50% probability. However, we can take the label of the pair, which was first picked up, deterministically; it is the same situation as the two-up two-down case of the Ising-model block-spin transformation^21^. It is noteworthy that the procedure of block-cluster transformation is the same for *q*-state models irrespective of *q*. In the framework of the dual Monte Carlo algorithm^34–36^, the block-cluster transformation is a transformation in the graph degrees of freedom, whereas the block-spin transformation is that in the spin degrees of freedom. It is instructive to compare the program code for the block-cluster transformation with that for the block-spin transformation. The essential part to select the label of the block cluster is 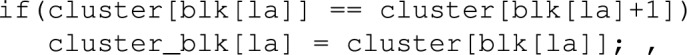 which is in parallel to the block-spin transformation: 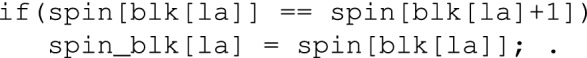 In the program code, $\texttt {cluster[]}$ stands for the label of the original cluster, whereas $\texttt {cluster\_blk[]}$ stands for that of the block cluster. The command means that if the two labels in the block coincide, we choose this label as the label of the block cluster.

### Detailed description of SR

We describe the detailed procedure of the SR with a deep CNN. It is an extension of the method by Efthymiou et al.^21^. The improved estimator of the correlation configuration is used instead of the spin configuration. Starting from $2L \times 2L$ spin system of improved correlation configuration, $\{ \xi _i \}$, we produce $L \times L$ system of improved correlation configuration, $\{ g_i \}$, using the block-cluster transformation. We aim to reproduce the $2L \times 2L$ target improved correlation configuration using the SR mapping of a supervised learning approach. This procedure is also illustrated in Fig. 6. The improved correlation configurations take a value of 1, 1/2, or 0 because we consider the average value of *x*- and *y*-directions. According to Ref. ^16^, they are represented in white, light gray or dark gray.

We consider three layers for the SR CNN, patch extraction and representation, non-linear mapping, and reconstruction^22^. The first layer is an upsampling layer by copying each configuration to $2 \times 2$ block. This upsampling procedure is appropriate both for low temperature and high temperature. It is in contrast to the situation of using spin configuration^21^, where a simple upsampling procedure is insufficient for random configuration of high-temperature side. For the improved correlation configuration, it takes $+1$ when the correlation length is as large as half of the system size, *L*/2, and the sites with $+1$ correlation form a small cluster (see Fig. 1f of Ref. ^16^). For a convolution layer, as the second layer, the transformation $f(W * x + b)$ is applied to the input *x*, a $2 \times 2$ improved correlation configuration. Here, *W* ($2 \times 2$) is the filter, *b* being the bias vector, and $*$ being the convolution operation. To avoid truncating the image edge, we add the periodic boundary padding. For activation function *f*, we use a sigmoid function, which gives the probability of each site For a loss function, we use the cross-entropy loss function between $\{ \xi _i \}$ and $\{ p_i \}$ (continuous variable);

4 $$\begin{aligned} L(\{\xi _i\}, \{p_i \}) = - \sum _{i=1}^{N} \Big [ \xi _i \cdot \ln p_i + (1 - \xi _i) \cdot \ln (1 - p_i ) \Big ], \end{aligned}$$

where $\cdot$ denotes the element-wise product between matrices. As a library we use “BCEWithLogitsLoss”, where BCE stands for binary cross entropy. Parameters ($\theta =(W,b)$) are tuned to minimize a loss function. We employ the Adam method^37^ as an optimizer. As the third layer, using the optimized parameters, we calculate each $\{ p_i \}$, and determine $+1$ or 0 depending upon this probability. Repeating this process two times, we emulate a configuration as the sum of *x*- and *y*-directions.

Thus, we can reproduce $\{ \xi _i' \}$ of $2L \times 2L$ size. Because we deal with the correlation, we can treat the three-state Potts model; the permutational symmetry is taken into account. When we consider the spin configuration, we cannot follow the present super-resolution procedure. We emphasize that the same procedure can be used for any *q*-state Potts model.
